# Genome-Wide Identification and Analysis of Glycosyltransferases in *Colletotrichum graminicola*

**DOI:** 10.3390/microorganisms12122551

**Published:** 2024-12-11

**Authors:** Yafei Wang, Honglian Li, Jiaxin Chang, Yu Zhang, Jinyao Li, Shaofeng Jia, Yan Shi

**Affiliations:** 1 College of Plant Protection, Henan Agricultural University, Zhengzhou 450002, China; honglianli@sina.com (H.L.); changjiaxin2104@163.com (J.C.); 18436618796@163.com (Y.Z.); lijinyao2412@163.com (J.L.); 2 Shenzhen Branch, Guangdong Laboratory for Lingnan Modern Agriculture, Genome Analysis Laboratory of the Ministry of Agriculture, Agricultural Genomics Institute at Shenzhen, Chinese Academy of Agricultural Sciences, Shenzhen 440307, China; 3 Syngenta (China) Investment Co., Ltd., Shanghai 200126, China; shaofeng.jia@syngentagroup.cn

**Keywords:** *Colletotrichum graminicola*, glycosyltransferase, gene family, expression pattern

## Abstract

Corn leaf blight and stem rot caused by *Colletotrichum graminicola* are significant diseases that severely affect corn crops. Glycosyltransferases (GTs) catalyze the transfer of sugar residues to diverse receptor molecules, participating in numerous biological processes and facilitating functions ranging from structural support to signal transduction. This study identified 101 *GT* genes through functional annotation of the *C. graminicola* TZ–3 genome. Subsequent analyses revealed differences among the *C. graminicola GT* (*CgGT*) genes. Investigation into subcellular localization indicated diverse locations of CgGTs within subcellular structures, while the presence of multiple domains in CgGTs suggests their involvement in diverse fungal biological processes through versatile functions. The promoter regions of *CgGT* genes are enriched with diverse cis-acting regulatory elements linked to responses to biotic and abiotic stresses, suggesting a key involvement of *CgGT* genes in the organism’s multi-faceted stress responses. Expression pattern analysis reveals that most *CgGT* genes were differentially expressed during the interaction between *C. graminicola* and corn. Integrating gene ontology functional analysis revealed that *CgGT*s play important roles in the interaction between *C. graminicola* and corn. Our research contributes to understanding the functions of *CgGT* genes and investigating their involvement in fungal pathogenesis. At the same time, our research has laid a solid foundation for the development of sustainable agriculture and the utilization of *GT* genes to develop stress-resistant and high-yield crop varieties.

## 1. Introduction

Protein glycosylation is a crucial process closely associated with the structure and functionality of proteins. In eukaryotic cells, glycosylation is a prevalent post-translational modification of numerous eukaryotic proteins [1,2,3]. Glycosylated eukaryotic proteins account for over half of all proteins [4]. Typically occurring after or during protein synthesis, glycosylation represents a highly diverse form of protein modification [5]. This modification can enhance protein solubility while reducing susceptibility to protein hydrolysis. Researchers have increasingly recognized the significance of protein glycosylation in processes such as protein localization, transport, and intercellular interactions.

Glycosyltransferases (GTs) are widely distributed in various organisms [6]. GTs are present in both prokaryotes and eukaryotes, with a significant proportion existing as Golgi membrane proteins in eukaryotic cells. These essential proteins help transfer sugar residues to receptor molecules, thereby mediating diverse biological functions [3,7,8,9]. GTs play an essential role in cell wall biosynthesis, cell adhesion, and cell signaling [10,11,12]. They play critical roles in synthesizing secondary metabolites and responding to both abiotic and biotic stresses. The metabolites are vital not only for the growth and development of organisms. but also for their ability to withstand pathogens, pests, and other biological stresses [13]. Moreover, GTs are crucial in mediating organisms’ responses to drought, salinity, and low temperatures. They regulate osmotic balance, antioxidant capacity, and signaling pathways by facilitating glycosylation reactions [14,15]. Furthermore, GTs play a key role in synthesizing osmoregulatory substances, such as proline and mannitol, which assist organisms in maintaining cellular hydration under drought and high salinity conditions [16]. Utilizing genetic engineering techniques, researchers can enhance or introduce specific *GT* genes to improve disease resistance, stress tolerance, and yield in crops [17]. This approach holds significant potential for applications in sustainable agriculture.

*Colletotrichum graminicola* is a severe pathogen that induces stem rot and leaf blight on corn, ranking among the most devastating diseases affecting corn [18,19,20]. This pathogen primarily impacts corn crops and leads to annual losses of up to $1 billion in the United States alone [19]. The worldwide prevalence of this disease poses a significant obstacle to maize cultivation. The lack of effective resistant varieties sustains a strong reliance on chemical pesticides for disease management, leading to unforeseen environmental consequences. Moreover, pesticide residues present risks to human and livestock health. The increasing resistance of pathogens to pharmaceutical treatments also reduces the effectiveness of chemical control methods. Considering the widespread cultivation of maize globally, there is a critical need for a sustainable and innovative solution to ensure the protection of maize production and food security. 

Recent advancements in genome sequencing, annotation, genome editing technologies, whole-genome association studies, genomic selection, and the integration of genomics and phenomics provide researchers with more efficient tools for breeding high-yielding, disease-resistant, and stress-tolerant maize varieties essential for ensuring food security. The application of transgenic technology can enhance plants’ ability to resist pathogens, with GTs playing a crucial role in this process. Research indicates that increasing the expression of specific *GT* genes can strengthen plant disease resistance, support plant cell wall formation, and improve plants’ ability to withstand various stress factors [21,22]. In recent years, researchers have placed a growing emphasis on studying the genome structure and pathogenic mechanisms of key fungal pathogens in maize. The availability of high-quality genomes of pathogenic fungi has facilitated the analysis of genes associated with crucial pathogenic processes [23]. The primary aims of this study are to identify and analyze the *GT* genes in *C*. *graminicola* and to examine their expression patterns during maize infection. This study enriches the available resources on *GT* genes and expands the application possibilities of *GT* genes in sustainable agriculture.

## 2. Materials and Methods

### 2.1. Identification and Analysis of GTs

The data for *C. graminicola* strain TZ-3 were acquired from publicly available genome sequencing databases [23]. The properties of GTs were predicted through ExPASy available at https://web.expasy.org/protparam/ (accessed on 28 September 2024) [24]. The online tool available at https://wolfpsort.hgc.jp/ (accessed on 28 September 2024) was used to analyze subcellular localization.

### 2.2. Phylogenetic Analysis

GT protein sequences from *C. graminicola* TZ-3 were analyzed using Clustal W in MEGA 5.0, followed by the construction of a phylogenetic tree in MEGA 5.0. The neighbor-joining method was utilized to construct the evolutionary tree, with a bootstrap value set at 1000 iterations. Subsequently, the tree underwent further refinement through the EVOLVIEW website (http://evolgenius.info//evolview-v2/) (accessed on 30 September 2024).

### 2.3. Gene Structures and Protein Motifs

We utilized the online tool available at https://gsds.gao-lab.org/index.php (accessed on 28 September 2024) to predict structures of *GT* genes [25]. The online tool available at http://meme-suite.org (accessed on 28 September 2024) was employed to analyze conserved motifs of GT proteins, using a motif count of 10 [26]. 

### 2.4. Identification of Cis-Acting Regulatory Elements (CAREs) and Gene Ontology (GO) Analysis

The 2000 bp upstream promoter sequences of *GT*s were extracted from *C. graminicola* TZ-3 [23]. The online tool available at http://bioinformatics.psb.ugent.be/webtools/plantcare/html/ (accessed on 28 September 2024) was employed to identify CAREs, while GO functional analysis was conducted using the online tool available at https://www.omicshare.com/tools (accessed on 28 September 2024).

### 2.5. Expression Pattern Analysis in GT Family

We obtained RNA-seq data of *C. graminicola* from prior studies and conducted an analysis to elucidate the expression changes of *GT* genes [20]. The online tool available at https://www.omicshare.com/tools (accessed on 30 September 2024) was utilized to generate heat maps using FPKM values.

### 2.6. RT-qPCR

RT-qPCR experiments were performed to validate the transcriptome data. Total RNA was extracted from maize infected with *C. graminicola* using the Tiangen DP441 assay kit (Tiangen, Beijing, China). Subsequently, cDNA was synthesized using a HiScript III first-strand cDNA synthesis kit (Vazyme, Nanjing, China). The analysis was conducted utilizing a SYBR qPCR Master Mix kit (Vazyme, Nanjing, China) and an ABI 7500 real-time system (Applied Biosystems, Foster, CA USA). The *UBQ* gene was chosen as the internal reference, and the results were determined using 2^−∆∆CT^.

## 3. Results

### 3.1. Identification and Physicochemical Properties Analysis of GTs in C. graminicola

All 101 *GT*s (named *CgGT1*–*CgGT101*) were found in the *C. graminicola* TZ–3 genome. Their detailed characteristics are listed in Table 1. The corresponding proteins consist of 238 to 2429 amino acids (aa) and have molecular weights ranging from 26.30 to 272.73 kDa. They can be categorized based on their isoelectric points (PI), with 35 CgGT proteins being alkaline (PI > 7.5), 50 being acidic proteins (PI < 6.5), and the remaining CgGT proteins being neutral. The grand average of hydropathicity (GRAVY) varies from –0.728 to 0.584. Among the CgGT proteins, 15 exhibit a GRAVY greater than 0, while the remaining proteins display a GRAVY less than 0. Subcellular localization analysis revealed that 43 CgGT proteins are found in the plasma membrane, 18 in the mitochondria, 18 in the extracellular space, 13 in the cytoplasm, 5 in the nucleus, 3 in the Golgi apparatus, and 1 in the endoplasmic reticulum (Table 1).

### 3.2. Phylogenetic Relationship

**Figure 1:**
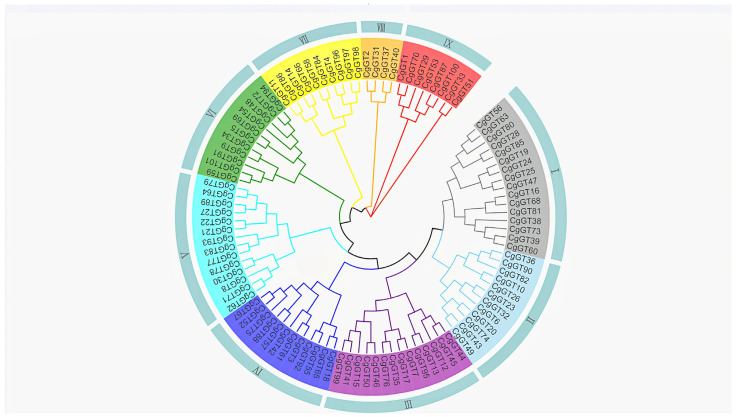
Phylogenetic tree of glycosyltransferases from *Colletotrichum graminicola*. Note: CgGTs are divided into nine groups (I–IX) and each color represents a group.

We constructed a phylogenetic tree using MEGA5 and analyzed the phylogeny of the CgGTs (Table S1). The results indicated that CgGTs were classified into nine groups, specifically Groups I to IX, comprising 16, 12, 14, 12, 14, 11, 10, 4, and 8 members, respectively (Figure 1). Certain groups exhibited closer genetic relationships, such as Groups I and II, while others, such as Groups I and IX, demonstrated more distant relationships.

### 3.3. Sequence and Structural Analysis of CgGTs

We conducted a comprehensive analysis of the structural characteristics of all *CgGT* genes (Figure S1, Table S1). The analysis revealed significant variations in the gene structure among the *CgGT* genes. Specifically, the *CgGTs* exhibited a range of 0 to 13 introns and 1 to 14 exons. Notably, *CgGT12* stood out, with the highest count of both exons and introns, whereas 18 *CgGTs* were intronless. Moreover, structural domain analysis revealed a diverse array of superfamily domains present in CgGT, such as the PRK14501, glycosyltransferase GTB-type, and PMT 2 superfamily domains, among others. Particularly noteworthy were CgGT6 and CgGT19, each harboring six distinct superfamily domains.

**Figure 2:**
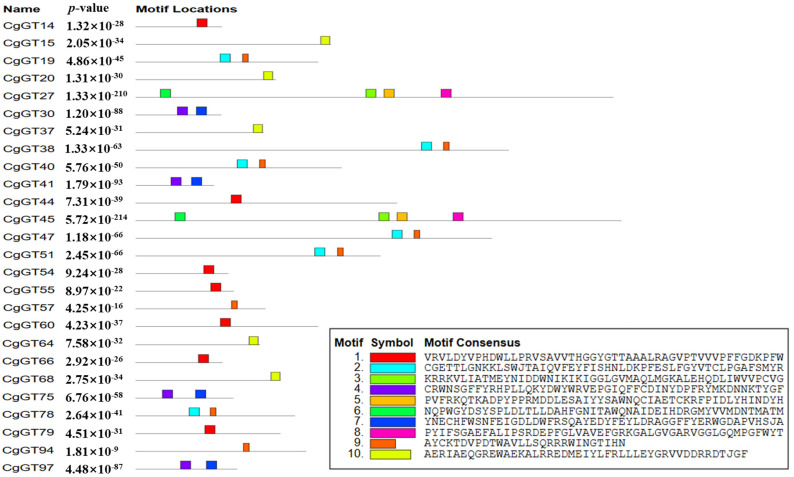
The motifs of CgGTs. Note: Boxes of different colors represent different conserved motifs.

To gain further insight into the functionality of the CgGTs, we analyzed conserved motifs within these CgGT proteins. Subsequently, we identified ten motifs in CgGT proteins (Figure 2 and Figure S2). Among these motifs, motif 9 stood out as the most prevalent, being present in eight CgGT proteins. Additionally, seven CgGT proteins exhibited motif 1, six contained motifs 2 and 9, five featured motif 10, and four showcased motifs 4 and 7. Furthermore, CgGT27 and CgGT45 shared four identical motifs, specifically motifs 3, 5, 6, and 8.

### 3.4. Sequence Analysis of CgGT Gene Family Promoters

**Figure 3:**
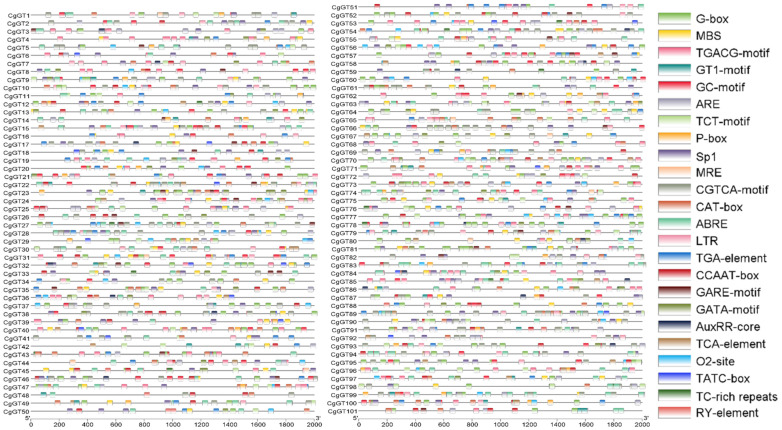
Cis-acting regulatory elements in the promoter regions of *CgGT* genes. Note: Different colored boxes represent different cis-acting regulatory elements.

**Figure 4:**
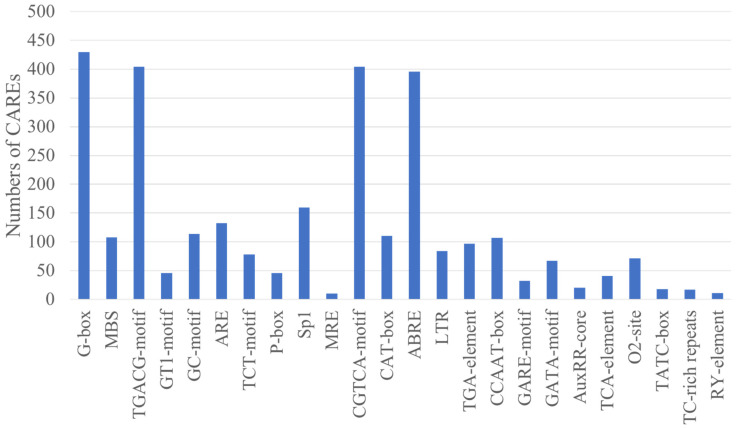
The numbers of predicted cis-acting regulatory elements in the promoter regions of *CgGT* genes.

The upstream promoter regions of *CgGT* genes were analyzed (Table S1). The promoter sequences of these *CgGT* genes contain various CAREs related to stress response, pathogenicity, and development (Figure 3, Table S2). Further analysis revealed that these promoter sequences predominantly contain the response elements associated with jasmonate, abscisic acid, salicylic acid, auxin, gibberellin, drought, low-temperature, defense stress, and light. Notably, methyl jasmonate (MeJA) response elements are the most abundant (808), followed by light response elements (791) and abscisic acid response elements (396) (Figure 4, Table S2). These findings suggest that CgGTs may have significant roles in responding to diverse stresses, as well as in fungal growth and pathogenicity. The *CgGT* genes also contain CAREs related to zein metabolism regulation, anoxic-specific inducibility, and anaerobic induction (Table S2). These results indicates that CgGTs may be associated with various biological processes.

### 3.5. GO Analysis of CgGTs

**Figure 5:**
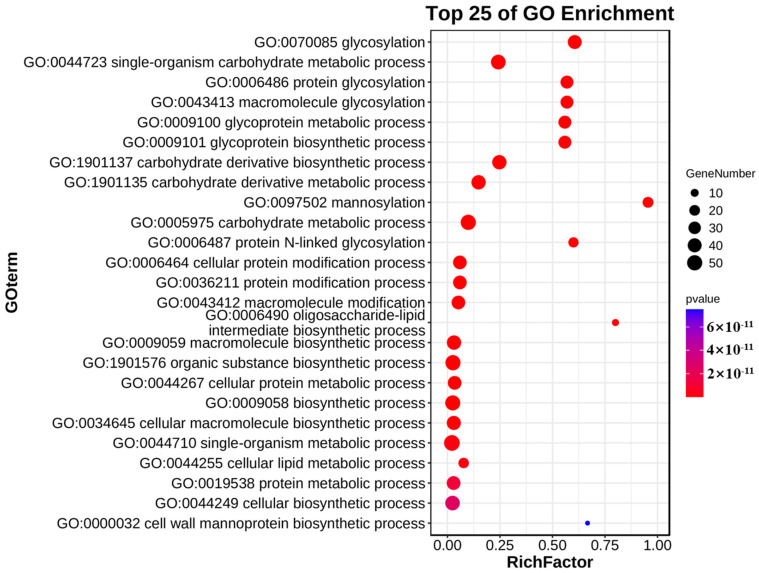
Gene ontology enrichment analysis of *CgGT* genes.

We conducted GO enrichment analysis on the *CgGT*s here. Our results revealed that *CgGT*s were significantly associated with multiple GO terms, including glycosylation (GO:0070085), monomer carbohydrate metabolic processes (GO:0044723), protein glycosylation (GO:0006486), and glycoprotein metabolic processes (GO:0009100), among others (Figure 5, Table S3). The research results indicate that *CgGTs* mainly contribute to glycosylation and metabolic processes.

### 3.6. The Response of CgGT Family Genes in the Infection Process of C. graminicola

**Figure 6:**
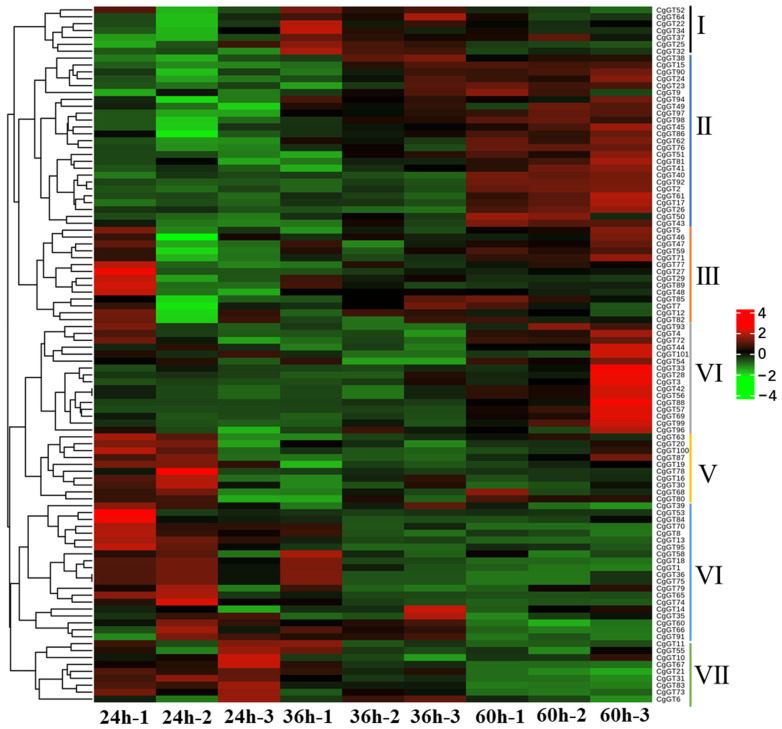
The expression level of *CgGTs* based on RNA-seq data. Note: *CgGT* genes were categorized into seven distinct classes (I–VII) based on expression profiles. Red and green indicate high and low expression levels of *CgGTs*, respectively.

**Figure 7:**
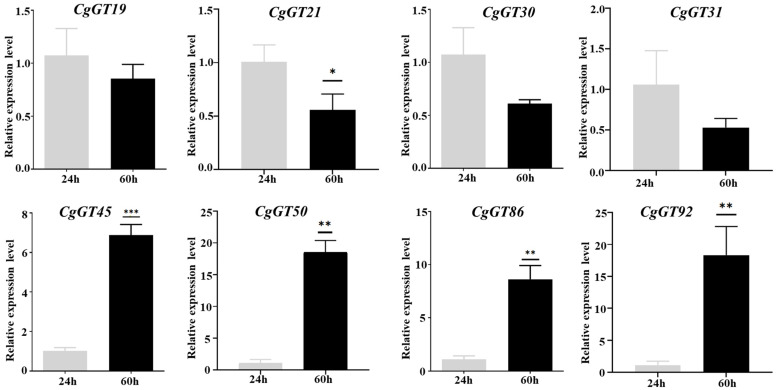
RT-qPCR verification of *CgGT* expression pattern. Note: * *p* < 0.05, ** *p* < 0.01, *** *p* < 0.001.

To investigate *CgGT* family genes associated with the pathogenesis of *C. graminicola*, RNA-seq data was used to analyze the expression changes of 101 *CgGT* genes during pathogen infection [20]. A heatmap was constructed based on FPKM values of the 101 *CgGT* family genes, illustrating their expression dynamics at three time points (24, 36, and 60 h) post-inoculation (Figure 6). Our findings revealed that based on expression profiles, the 101 *CgGT* family genes could be categorized into seven distinct classes. In Class I, consisting of 7 *CgGTs*, gene transcription initially increased and then decreased during infection. Class II comprised 25 *CgGTs*, while Class IV included 16 *CgGTs*, showing a progressive upregulation in transcription levels throughout the infection process. Notably, Class VI and VII encompassed a total of 29 *CgGTs*, with their transcription levels gradually decreasing during infection. Eight *CgGT* genes were randomly chosen for RT-qPCR validation, and primers were designed accordingly (Table S4). The RT-qPCR validation results confirmed the reliability of the transcriptome data (Figure 7). These *CgGT* family genes exhibited substantial alterations in expression patterns during pathogen infection, suggesting their potential involvement in the pathogenic mechanisms of the pathogen.

## 4. Discussion

GTs are essential for numerous fundamental biological processes, mediating many functions [3,7,8]. These enzymes catalyze sugar transfer reactions, transferring sugar moieties from substrate molecules to another molecule. GTs are involved in many biosynthetic pathways, including the synthesis and modification of secondary metabolites and cell walls [27,28]. Moreover, *GT* genes regulate organisms’ responses to various stresses, contributing to the synthesis of polysaccharides in biological cell walls. As structural polysaccharides are crucial for organisms’ growth and stress resistance, the regulation of *GT* genes can improve the stability of biological cell membranes and enhance tolerance to adversity. Research conducted in the past has demonstrated the capability of a barley UDP-glucose GT, known as HvUGT13248, to efficiently detoxify deoxynivalenol, a toxin produced by *Fusarium graminearum* [22]. Expression of this enzyme in transgenic wheat results in a significant type II resistance response against fungi that produce deoxynivalenol, offering a promising strategy for mitigating *Fusarium* head blight in wheat. Considering the pivotal role of GTs in plants’ defense mechanisms against pathogen infiltration, the numerous *GT* genes pinpointed in this investigation could serve as valuable assets for the development of resilient maize cultivars that promote sustainable agricultural practices.

We identified GTs in the *C. graminicola* TZ-3 genome and analyzed their characteristics in this study. The analysis results showed that most CgGT proteins were hydrophilic, and nearly half of CgGT proteins were acidic. Phylogenetic analysis revealed that 8 CgGTs in group IX exhibited distant relationships with CgGTs in other groups, suggesting potential significant structural and functional variances compared to other CgGTs. The phylogenetic analysis demonstrates that CgGTs sharing similar structural domains tend to cluster together, while certain CgGTs cluster in distinct branches, potentially indicating functional diversity. The diverse nature of fungal GTs expands the scope of their potential applications. The correlation between the number of introns with gene function suggests that a lower intron count can enhance gene activation speed [29,30,31]. Discrete features characterize the distribution pattern of introns in microbial genes [32]. Among the *CgGT* genes, *CgGT12* possesses 13 introns, while 18 *CgGTs* lack introns, indicating potential variations in intron numbers within *CgGT* genes throughout the evolutionary processes.

Subcellular localization analysis reveals that the CgGT proteins exhibit diverse distribution patterns across various cellular compartments. Among the 43 CgGT proteins identified in the plasma membrane, their presence suggests potential involvement in intercellular interactions and membrane architecture. The 18 CgGT proteins found extracellularly may either undergo secretion or be transported outside the cell membrane to exert their functional effects. Moreover, the 18 CgGT proteins localized within the mitochondria are likely associated with mitochondrial metabolic activities and structural integrity. In the cytoplasm, the presence of 13 CgGT proteins indicates a role in influencing cellular functions, growth, and metabolism. Additionally, five CgGT proteins are situated within the nucleus, suggesting their involvement in nuclear structure maintenance and genetic information processing. Furthermore, through bioinformatics analysis, we identified a diversity of protein domains within CgGTs, indicating their potential multifunctionality. The varied subcellular distribution of CgGT proteins implies their participation in fungal biological processes through multiple pathways, influencing the interactions between pathogenic fungi and their hosts. These results are consistent with previous reports highlighting the versatile roles of GTs in mediating various biological functions, from structural maintenance to signal transduction, thereby playing a crucial role in numerous cellular processes [7,8]. 

The CAREs present in promoter regions exert noteworthy influences on gene functionality [33]. GTs are pivotal for the biosynthesis of various secondary metabolites, crucial for organism development and resilience against biological stressors [13]. Unsurprisingly, we identified CAREs in *CgGT* promoter regions associated with stress and defense response. We also identified CAREs related to growth and light responsiveness. GTs are related to plant hormone synthesis and modification, thereby regulating plant growth and development. The ABRE regulatory elements are also related to abiotic stress responses [34,35]. CAREs associated with plant hormones and developmental processes were also found in *CgGT* promoter regions. Numerous CAREs related to various plant hormones, including elements for MeJA, abscisic acid, gibberellin, and auxin responses, were detected. GTs are essential for mediating biological responses to salinity, drought, and low temperatures [14,15]. They also play an important role in synthesizing osmotic adjustment substances that aid in cellular hydration maintenance in drought-prone and high-salinity environments [16]. Several CAREs associated with low-temperature response, stress response, and defense mechanisms were also identified. Additionally, many CAREs are closely linked to gene family functions [36,37,38,39,40,41,42]. *GT* genes are characterized by abundant CAREs that are indispensable for stress responses, offering a promising avenue for the creation of maize varieties resistant to diseases. The findings of this study align with prior research [21]. Previous studies have highlighted the essential role of the maize glycosyltransferase UFGT2 in modifying flavonols, thereby enhancing plant resilience to abiotic stress. Mutants with knocked-out *ufgt2* genes exhibit marked sensitivity to salt and drought stress. These *ufgt2* mutants show a notable decrease in total flavonol levels and reduced ability to scavenge H_2_O_2_. 

The results of the GO analysis for *CgGT* genes indicate their primary involvement in glycosylation and metabolic processes, particularly in the metabolism of monomeric carbohydrates and glycoproteins. Glycosylation modifications are critical for biological development and are likely associated with the roles of glycoproteins in cellular recognition and signal transduction [43,44]. Glycoproteins serve multiple essential functions in organisms, including cellular recognition, signal transduction, cell adhesion, and immune response [44]. They play a vital role in host-microbe interactions. These analytical findings suggest that *CgGT* genes are implicated in pathogen–host interactions and are closely associated with pathogenic mechanisms. The functions of these genes suggest that they may contribute to the creation of resistant maize varieties, thereby reducing the use of chemical pesticides and promoting the development of sustainable agriculture.

Moreover, we investigated the changes in *CgGT* gene expression levels during the infection of maize hosts by *C. graminicola*. The function of genes is closely related to their expression characteristics [45,46,47]. Our analysis revealed varying levels of differential expression of *CgGT* genes at different stages of pathogen infection, implicating their involvement in pathogen–host interactions. The heatmap suggests that certain *CgGT* genes are primarily associated with the initiation of fungal infections, showing a gradual decrease in transcription levels as the infection progresses. Conversely, some genes seem to contribute to lesion enlargement, as evidenced by an increase in their transcription levels over time. Additionally, certain genes may affect the intermediate stage of fungal infection, displaying an initial increase and subsequent decrease in transcription levels with infection progression. These findings underscore the diverse roles of *CgGT* genes across different infection time points, indicating functional variability. Overall, these genes are involved in pathogen infection and disease expansion. Their important role in the interaction between pathogens and hosts will help to apply them to crop disease resistance research and provide ideas for sustainable agricultural construction.

Whole genome analysis of the *GT* genes provides a foundation for studying the function of *CgGTs*. It is important to note that the conclusions drawn in this article are based on bioinformatics analysis. The subsequent reactions following the expression of these genes in *C. graminicola* remain unclear. Future research should focus on the interactions between *CgGTs* and host-triggered genes. Furthermore, *GT* genes are crucial in sustainable agriculture as they confer resistance to biotic and abiotic stresses. This study offers a comprehensive analysis of *GT* genes in *C. graminicola*, enriching the available *GT* gene resources. By thoroughly investigating the functions and regulatory mechanisms of these genes, researchers can aid in developing stress-tolerant, high-yield crop varieties. Understanding the characteristics and functions of *GT* genes will enable deeper exploration of their potential applications in agriculture, thereby advancing sustainable agricultural practices. Through genetic engineering, researchers can enhance or integrate specific *GT* genes to improve crops’ resilience to biotic and abiotic stresses, consequently reducing reliance on pesticides and fertilizers.

## 5. Conclusions

The study conducted a detailed analysis of the *CgGT* genes and their expression patterns during pathogen infection, enhancing our understanding of this gene family. The research identified 101 *GT* genes in the *C. graminicola* TZ-3 genome, revealing their presence in various subcellular structures. These *CgGT* genes contain multiple conserved domains, indicating diverse functions and involvement in fungal biological processes that affect the interaction between pathogenic fungi and hosts. Promoter regions of these genes are rich in conserved CAREs associated with biotic and abiotic stress responses, suggesting their role in the organism’s stress response. Expression analysis demonstrated differential expression of most *CgGT* genes during pathogen–host interactions, with functional analysis highlighting their involvement in host–microbe interactions and the pathogenic mechanisms of pathogens. This study not only enriches *GT* gene resources but also presents new opportunities for leveraging *GT* genes in sustainable agriculture.

## Figures and Tables

**Table 1:** Characteristics of putative glycosyltransferases in *Colletotrichum graminicola*.

Proposed Gene Name	Gene ID	CDS Length (bp)	Protein Length (aa)	Mw (KDa)	pI	GRAVY	Predicted Subcellular Localization
CgGT1	EVM0000312	1119	372	42.13	6.73	−0.221	extracellular, including cell wall
CgGT2	EVM0000372	2142	713	81.25	6.05	−0.448	cytosol
CgGT3	EVM0000427	1014	337	38.21	6.31	−0.313	extracellular, including cell wall
CgGT4	EVM0000494	2244	747	82.13	5.77	−0.728	nucleus
CgGT5	EVM0000520	5823	1940	221.5	7.49	−0.156	plasma membrane
CgGT6	EVM0000577	4428	1475	164.95	5.08	−0.286	plasma membrane
CgGT7	EVM0000595	2247	748	83.70	8.94	0.207	plasma membrane
CgGT8	EVM0000840	1566	521	58.41	8.01	−0.019	mitochondrion
CgGT9	EVM0000878	717	238	26.58	8.79	−0.104	mitochondrion
CgGT10	EVM0001034	1659	552	61.65	9.04	−0.142	cytosol
CgGT11	EVM0001072	1341	446	49.09	5.77	−0.052	cytosol
CgGT12	EVM0001510	5289	1762	199.24	6.85	−0.414	cytosol
CgGT13	EVM0001638	1137	378	44.07	6.68	−0.425	extracellular, including cell wall
CgGT14	EVM0001682	1296	431	46.30	5.43	0.028	cytosol
CgGT15	EVM0001689	2931	976	109.09	6.71	−0.022	plasma membrane
CgGT16	EVM0001718	1563	520	59.60	6.29	−0.578	extracellular, including cell wall
CgGT17	EVM0001804	1230	409	46.03	5.95	−0.189	extracellular, including cell wall
CgGT18	EVM0001965	1302	433	49.34	9.18	−0.012	mitochondrion
CgGT19	EVM0002001	2742	913	103.24	6.13	−0.172	plasma membrane
CgGT20	EVM0002094	2106	701	78.52	7.03	−0.525	mitochondrion
CgGT21	EVM0002104	1596	531	61.21	8.26	−0.510	plasma membrane
CgGT22	EVM0002238	1104	367	41.31	7.75	−0.380	extracellular, including cell wall
CgGT23	EVM0002239	1743	580	62.32	9.66	0.430	plasma membrane
CgGT24	EVM0002267	2217	738	83.80	7.38	−0.011	plasma membrane
CgGT25	EVM0002284	1293	430	48.38	9.47	0.511	plasma membrane
CgGT26	EVM0002503	1383	460	53.15	5.31	−0.468	extracellular, including cell wall
CgGT27	EVM0002506	7173	2390	264.75	5.71	−0.164	plasma membrane
CgGT28	EVM0002539	2247	748	83.78	6.40	−0.314	mitochondrion
CgGT29	EVM0002695	2205	734	83.17	9.28	0.203	mitochondrion
CgGT30	EVM0002790	1287	428	50.20	5.89	−0.724	Golgi apparatus
CgGT31	EVM0002951	1584	527	58.45	6.17	−0.307	extracellular, including cell wall
CgGT32	EVM0003094	1266	421	47.71	7.71	−0.271	mitochondrion
CgGT33	EVM0003195	1179	392	45.80	6.02	−0.564	mitochondrion
CgGT34	EVM0003225	1803	600	67.31	9.54	0.381	plasma membrane
CgGT35	EVM0003259	1809	602	69.15	9.70	−0.719	plasma membrane
CgGT36	EVM0003732	1209	402	45.63	7.67	−0.023	cytosol
CgGT37	EVM0003755	1920	639	72.94	8.81	−0.531	mitochondrion
CgGT38	EVM0003893	5601	1866	207.16	6.65	−0.204	plasma membrane
CgGT39	EVM0003945	1200	399	43.58	6.87	−0.011	plasma membrane
CgGT40	EVM0003981	3093	1030	116.35	6.17	−0.258	plasma membrane
CgGT41	EVM0004307	1182	393	46.50	5.76	−0.637	extracellular, including cell wall
CgGT42	EVM0004462	1494	497	56.99	6.09	−0.176	Golgi apparatus
CgGT43	EVM0004588	1233	410	45.47	8.94	−0.391	plasma membrane
CgGT44	EVM0004712	3927	1308	141.69	5.69	−0.447	cytosol
CgGT45	EVM0004723	7290	2429	272.73	6.30	−0.216	plasma membrane
CgGT46	EVM0004757	1551	516	59.07	9.42	0.272	plasma membrane
CgGT47	EVM0004896	5352	1783	197.36	5.54	−0.179	plasma membrane
CgGT48	EVM0004972	4824	1607	177.57	8.27	−0.373	nucleus
CgGT49	EVM0004975	3792	1263	140.20	7.16	−0.328	plasma membrane
CgGT50	EVM0005005	1509	502	57.91	9.00	−0.173	plasma membrane
CgGT51	EVM0005211	3675	1224	137.19	8.82	−0.303	plasma membrane
CgGT52	EVM0005219	1626	541	59.93	9.05	−0.034	mitochondrion
CgGT53	EVM0005273	1335	444	50.51	6.50	−0.422	mitochondrion
CgGT54	EVM0005326	1395	464	49.50	5.77	−0.018	mitochondrion
CgGT55	EVM0005437	1476	491	54.50	5.89	−0.157	mitochondrion
CgGT56	EVM0005573	1611	536	58.65	5.85	−0.167	extracellular, including cell wall
CgGT57	EVM0005623	1950	649	74.18	8.77	−0.039	plasma membrane
CgGT58	EVM0005717	1437	478	53.55	8.74	−0.056	plasma membrane
CgGT59	EVM0005768	1482	493	56.50	5.88	−0.605	nucleus
CgGT60	EVM0005789	2745	914	100.18	9.22	−0.264	plasma membrane
CgGT61	EVM0005882	1599	532	59.83	5.48	−0.261	cytosol
CgGT62	EVM0006207	1428	475	52.69	5.54	−0.356	extracellular, including cell wall
CgGT63	EVM0006283	1878	625	70.68	9.46	0.116	plasma membrane
CgGT64	EVM0006286	1863	620	71.52	6.78	−0.642	Golgi apparatus
CgGT65	EVM0006578	1596	531	59.56	5.76	−0.334	extracellular, including cell wall
CgGT66	EVM0006633	1299	432	45.57	6.14	0.011	mitochondrion
CgGT67	EVM0006833	903	300	33.55	6.52	0.158	plasma membrane
CgGT68	EVM0006930	2169	722	82.17	5.73	−0.289	plasma membrane
CgGT69	EVM0007087	2712	903	102.80	6.00	−0.486	nucleus
CgGT70	EVM0007165	1302	433	49.72	5.71	−0.313	extracellular, including cell wall
CgGT71	EVM0007204	1215	404	46.01	6.37	−0.402	mitochondrion
CgGT72	EVM0007220	3060	1019	114.74	5.97	−0.471	nucleus
CgGT73	EVM0007383	1662	553	62.31	6.17	−0.386	plasma membrane
CgGT74	EVM0007715	1338	445	50.67	6.17	−0.333	extracellular, including cell wall
CgGT75	EVM0007775	1461	486	55.29	5.55	−0.492	extracellular, including cell wall
CgGT76	EVM0007855	1485	494	55.72	5.39	−0.199	extracellular, including cell wall
CgGT77	EVM0008052	942	313	35.94	9.08	−0.396	plasma membrane
CgGT78	EVM0008073	2382	793	89.93	8.97	−0.074	plasma membrane
CgGT79	EVM0008174	2166	721	78.61	7.96	−0.026	plasma membrane
CgGT80	EVM0008429	1407	468	51.59	8.23	0.039	extracellular, including cell wall
CgGT81	EVM0008593	1119	372	42.42	5.63	−0.373	Endoplasmic reticulum
CgGT82	EVM0008674	1314	437	47.13	9.14	0.584	plasma membrane
CgGT83	EVM0008963	912	303	33.71	7.13	−0.159	mitochondrion
CgGT84	EVM0009050	1386	461	51.97	7.26	−0.378	mitochondrion
CgGT85	EVM0009152	2322	773	88.16	8.86	−0.089	plasma membrane
CgGT86	EVM0009253	2856	951	106.49	6.64	−0.195	plasma membrane
CgGT87	EVM0009723	2652	883	99.22	5.57	0.019	plasma membrane
CgGT88	EVM0009853	2067	688	77.57	7.88	−0.041	plasma membrane
CgGT89	EVM0010072	723	240	26.30	5.06	−0.128	mitochondrion
CgGT90	EVM0010156	1803	600	67.92	8.95	0.156	plasma membrane
CgGT91	EVM0010249	1083	360	41.40	8.15	−0.432	plasma membrane
CgGT92	EVM0010269	2664	887	100.26	5.62	−0.346	cytosol
CgGT93	EVM0010555	1152	383	43.46	4.85	−0.213	cytosol
CgGT94	EVM0010706	2547	848	95.61	6.42	−0.239	plasma membrane
CgGT95	EVM0010719	1452	483	54.16	6.20	0.067	cytosol
CgGT96	EVM0010739	1020	339	38.34	5.37	−0.518	cytosol
CgGT97	EVM0010808	1521	506	58.49	5.98	−0.477	plasma membrane
CgGT98	EVM0010822	7056	2351	262.09	6.01	−0.176	plasma membrane
CgGT99	EVM0011166	3807	1268	137.48	8.75	−0.602	cytosol
CgGT100	EVM0011280	2712	903	101.02	5.57	−0.034	plasma membrane
CgGT101	EVM0011800	1155	384	43.20	6.97	−0.264	extracellular, including cell wall

ID: identity; bp: base pair; aa: amino acids; KDa: kilo dalton; pI: isoelectric point; Mw: molecular weight; GRAVY: grand average of hydropathicity.

## Data Availability

The original contributions presented in the study are included in the article/Supplementary Materials, further inquiries can be directed to the corresponding authors.

## Supplementary Materials

The following supporting information can be downloaded at: https://www.mdpi.com/article/10.3390/microorganisms12122551/s1, Table S1. CgGT genomic, CDS, protein and promoter sequences. Table S2. Cis-acting regulatory elements are present in the *CgGT* gene promoter regions. Table S3. Gene ontology enrichment analysis of *CgGT* genes. Table S4. The primers used in this study. Figure S1. The gene structure and domain analysis of CgGTs. Note: (A) Structures of *CgGT* genes. (B) Domains of CgGTs. Figure S2. The sequences of CgGT proteins’ conserved motifs.
