# Supplementary material for: Genome-Wide Identification and Analysis of Glycosyltransferases in Colletotrichum graminicola
Source: Microorganisms. 2024 Dec 11;12(12):2551. doi: 10.3390/microorganisms12122551 (PMC11676771; doi:10.3390/microorganisms12122551)
Supplement: Supplementary file 1 [file microorganisms-12-02551-s001.zip › Figures S1 and S2.pptx]

## Slide 1
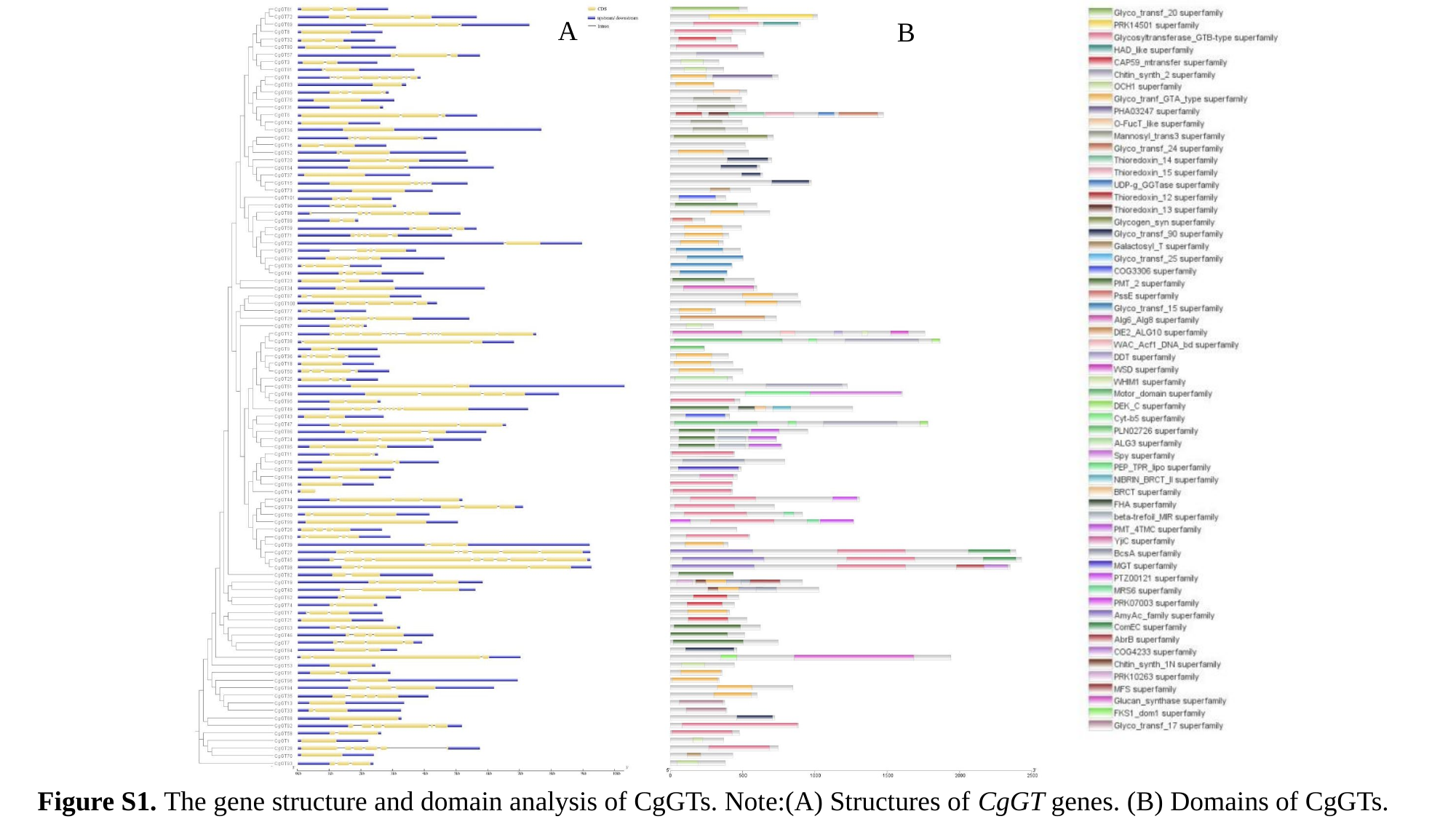

A
B
Figure S1. The gene structure and domain analysis of CgGTs. Note:(A) Structures of CgGT genes. (B) Domains of CgGTs.

## Slide 2
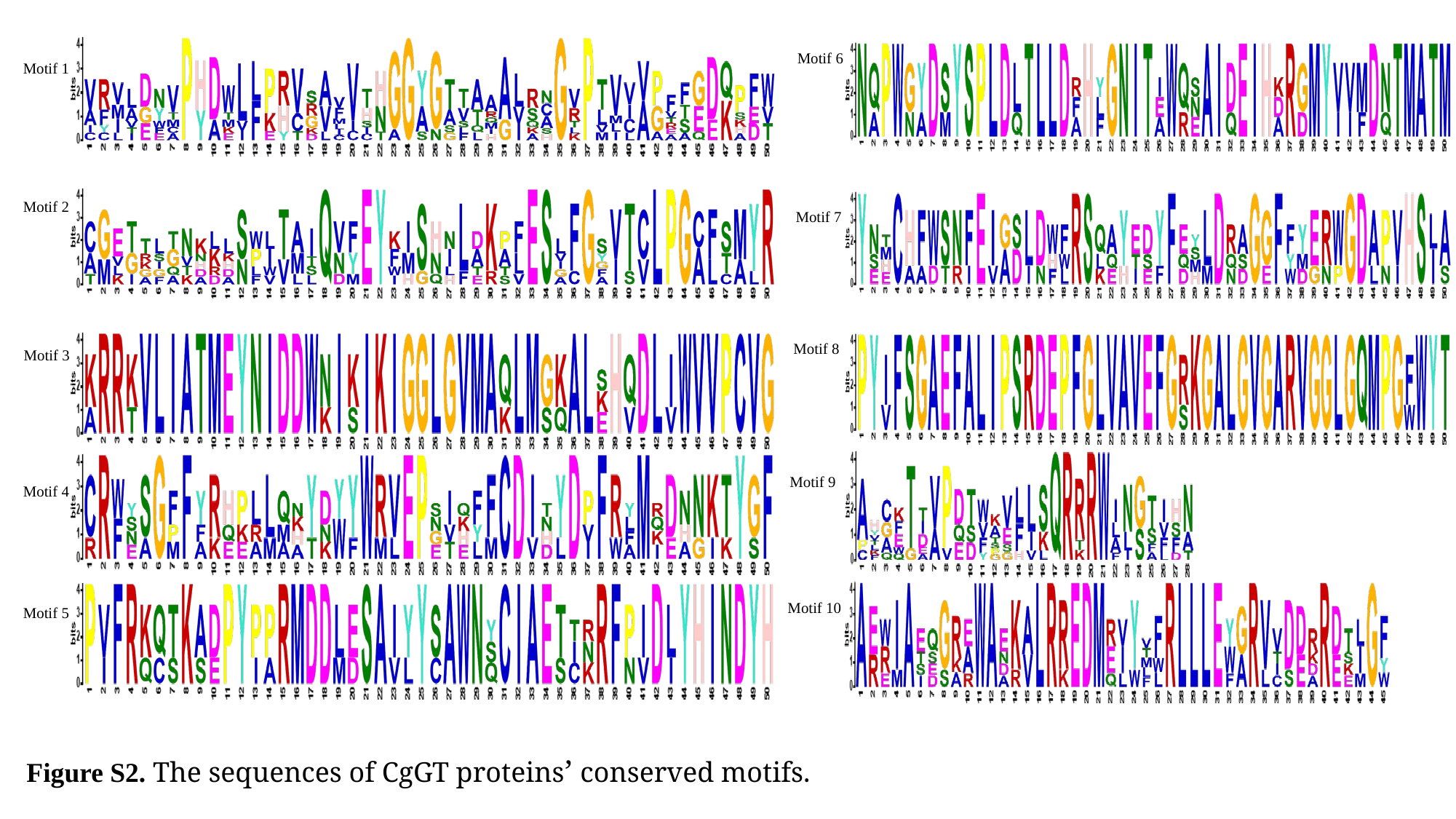

Motif 6
Motif 1
Motif 2
Motif 7
Motif 8
Motif 3
Motif 9
Motif 4
Motif 10
Motif 5
Figure S2. The sequences of CgGT proteins’ conserved motifs.
